# Getting on in Old Age: How the Gut Microbiota Interferes With Brain Innate Immunity

**DOI:** 10.3389/fncel.2021.698126

**Published:** 2021-07-06

**Authors:** Omar Mossad, Thomas Blank

**Affiliations:** ^1^Faculty of Medicine, Institute of Neuropathology, University of Freiburg, Freiburg, Germany; ^2^Faculty of Biology, University of Freiburg, Freiburg, Germany

**Keywords:** gut micobiota, microglia, inflammaging, senescence, metabolites, bacteria, brain, innate immunity

## Abstract

The immune system is crucial for defending against various invaders, such as pathogens, cancer cells or misfolded proteins. With increasing age, the diminishing immune response, known as immunosenescence, becomes evident. Concomitantly, some diseases like infections, autoimmune diseases, chronic inflammatory diseases and cancer, accumulate with age. Different cell types are part of the innate immunity response and produce soluble factors, cytokines, chemokines, and type I interferons. Improper maturation of innate immune cells or their dysfunction have been linked to numerous age-related diseases. In parallel to the occurrence of the many functional facets of the immune response, a symbiotic microbiota had been acquired. For the relevant and situation-dependent function of the immune system the microbiome plays an essential role because it fine-tunes the immune system and its responses during life. Nevertheless, how the age-related alterations in the microbiota are reflected in the innate immune system, is still poorly understood. With this review, we provide an up-to-date overview on our present understanding of the gut microbiota effects on innate immunity, with a particular emphasis on aging-associated changes in the gut microbiota and the implications for the brain innate immune response.

## Introduction

One of biology’s fascinations is the inevitability of death. However, human longevity has never been at the focal point as recently with the advances in socioeconomics, life sciences and medicine. With the increasing proportion of older individuals, comes the burden of maintaining a healthy lifespan. Aging affects multiple organ systems and processes, and increases the risk for neurodegenerative diseases like Alzheimer disease (AD) and Parkinson disease (PD) (Hou et al., 2019). Alterations in immune system functions and defense mechanisms is well described in aged individuals (Dorshkind et al., 2009). Therefore, the ability to delay or reverse the effects of aging on the immune system would have significant advantages. The cardinal features of the immune system are categorized into innate and adaptive functions.

A key difference between innate and adaptive immune cells can be seen when it comes to antigen recognition. The main reason is that the different cell types most likely had to fulfill specific tasks to fight cell death, tumorgenesis or infections. The different cell types responsible for the innate immune response comprise dendritic cells (DCs), macrophages, basophils, neutrophils, natural killer (NK) cells, eosinophils, and monocytes. Although it was long believed that the innate immune cells like macrophages and DCs show a rather “broad-spectrum” mode of recognition, they detect microbes with the help of pattern recognition receptors (PRRs) (Janeway, 1989). As an example, lipopolysaccharides (LPS) are part of the bacterial cell wall and can be sensed by Toll-like receptor 4 (Park and Lee, 2013). In the case of tissue damages or even cell death as a consequence of trauma or infection, so called “danger associated molecular patterns” (DAMPs) are recognized by other receptor-types. Among others, these molecular structures are presented by uric acid, high mobility group box 1 protein (HMGB1), heat shock proteins (Kono and Rock, 2008), and tumor cell DNA, which activates the stimulator of interferon genes (STING) pathway (Ohkuri et al., 2014; Woo et al., 2014). In addition, the expression of regulatory receptors on NK cells is modulated by other target cells and more specifically their expression level of the major histocompatibility complex (MHC) (Yokoyama, 2005). The primary innate immune cells of the brain are microglia. Microglia resembles tissue-resident macrophages and protect the brain by their reaction to potentially dangerous signals for the CNS cells, they remove cellular debris by phagocytosis, produce various types of cytokines to influence the microenvironment and can contribute to neuronal survival (Nayak et al., 2014). Importantly, innate cell receptors are germline-encoded and heritable, which is not the case for the antigen receptors of adaptive immunity. In this way, cells of the innate immune system are in the first line of defense when it comes to infections or tissue injury.

Multifaceted bacterial communities that colonize different body sites, represent an evolutionarily adjusted ecosystem, which holds an enormous multiplicity of genes that provoke direct interactions with physiological functions (Bischoff, 2016). One of the critical implications is most likely the regulation of the host immune system efficiency. When we look at the human digestive tract, the estimated total number of microorganisms in the intestine equals about the estimated total number of human somatic cells in the human body (Sender et al., 2016). Bacteria make up the highest number of all gut microbiota with about 500–1,000 different bacterial species (Sommer and Backhed, 2013). Bacteroidetes and Firmicutes are the most common bacterial phyla and make up about 90% of the whole intestinal microbiota (Qin et al., 2010). The remnant contains numerous species of other phyla. Although these species are only present in lower number, they might still produce paramount metabolites for salubrious aging. The progression of aging involves an imbalance in the status of the immune system (Nikolich-Žugich, 2018), where the microbiota might be playing a principal role in fine-tuning pro-inflammatory and anti-inflammatory activities of the immune cells (Zheng et al., 2020).

Various reports have described the interaction of the microbiota with brain function during the aging process but the centerpiece of the current review will be the modulation of the innate immune compartment by microbiota.

## Innate Immunity and Brain Aging

### Microglia

**FIGURE 1:**
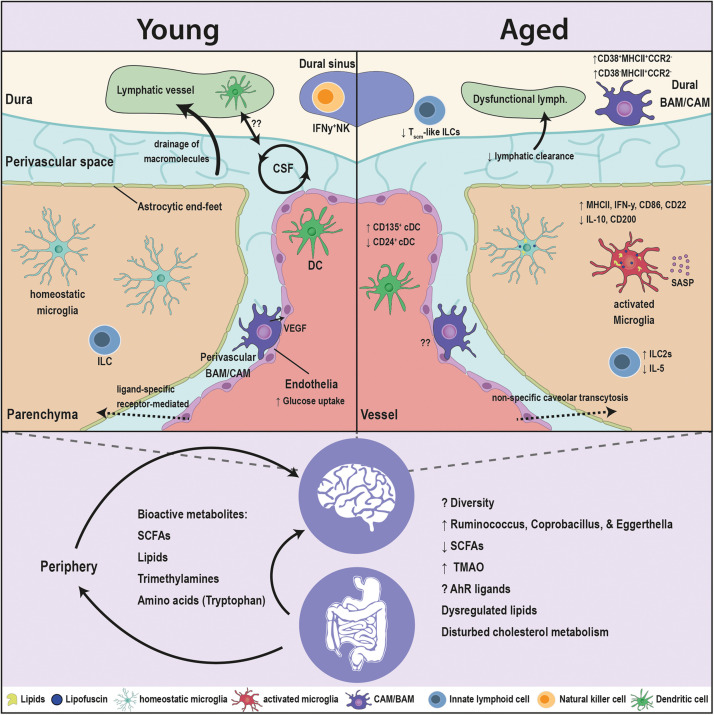
Age-associated alterations in the brain innate immunity and the gut microbiota. Major crippling and life-threatening diseases, such as cancer, cardiovascular disease, and neurodegeneration, are all linked to advanced age. The innate immune cells are the first responders to insults in the brain, and are in constant communication with the microbiota. Aging has several facets on the brain, as well as the microbiota. The blood brain barrier (BBB) switches from ligand-receptor transport to a less specific caveolar transcytosis. The drainage of the CSF macromolecules declines and is associated to dysfunctional changes in the lymphatics and innate immune cells. Microglia, the brain resident macrophages, switch to an age-related phenotype in which microglia accumulate lipofuscin and lipid droplets, elicit a pro-inflammatory profile [elevation in major histocompatibility complex (MHC) class II, interferon gamma (IFN-γ), cluster of differentiation 86 (CD86), and cluster of differentiation 22 (CD22), and a reduction in interleukin-10 (IL-10) and cluster of differentiation 200 (CD200)], decreased phagocytic capacity and an induction of senescence-associated secretory phenotype (SASP). CNS- or border-associated macrophages (BAMs/CAMs) undergo numerous brain-maintaining functions in the healthy young brain that decline with age. BAMs/CAMs shit to CD38^+^MHCII^+^CCR2^–^ and CD38^–^MHCII^+^CCR2^–^ subsets in the aged brain. Classical dendritic cells (DCs) associated with the brain elicit an elevation in CD135^+^ and a drop in CD24^+^ subsets. Innate lymphoid cells (ILCs) switch from IL1C to IL2C transcriptomic profile and lessen the expression of the neuroprotective interleukin 5 (IL-5). Moreover, the microbiota interacts with cell populations of the brain both directly and indirectly through modulating the periphery. Multitudes of microbiota-derived molecules [short chain fatty acids (SCFAs), lipids, trimethylamines, amino acids] influence the activity of the brain innate immune cells and in turn modulate the brain functions. The levels of these bioactive metabolites are sensitive to age. The fine tuning of these molecules is disturbed in aging, due to compositional changes in the microbiota and the drop in the taxonomic diversity. Alterations in the microbiota are decisive to the dysregulation of immune function of the brain’s innate immunity in aging and requires more comprehensive research.

Accounting for ∼15% of brain cellularity, the most critical innate immune cell for maintaining normal brain function is microglia (Figure 1). Microglia constantly monitor their environment with their cell processes while the soma remains rather stationary. While microglia mainly use their cellular processes for monitoring, they can also use them to establish cell-cell contact to neighboring cells and blood vessels (Nimmerjahn et al., 2005). In the cortex of aged individuals, microglial processes are changing in morphology and display a more fragmented and spheroid phenotype (Streit et al., 2004). These morphological changes can indicate cell activation. However, additional research results suggest that the perturbed appearance of microglia during the aging process is rather indicative of dystrophic and senescent cells (Sheng et al., 1998; Miller and Streit, 2007). The age-associated shortening of microglial cell processes and the downregulation of the microglial sensome genes cause a reduced coverage of brain parenchyma. It would be interesting to determine whether microglial age-related changes in morphology are responsible for a reduced cell-cell interaction of microglia with neurons and astrocytes (Hickman et al., 2013). Besides morphological changes of microglia during aging, they further display characteristic age-dependent lipofuscin granules and modified cytoplasmic granularity (Moreno-Garcia et al., 2018; Singh Kushwaha et al., 2018). Lipofuscin accumulates in the cytoplasm, most likely in the lysosomes, and is detectable as autofluorescent granules, which consist of oxidized macromolecules and metal ions. Since microglia cells that contain lipofuscin are often seen in close contact to neurons, we might assume that neurons secrete or actively transport lipofuscin toward microglia, which will then take it up (Brunk and Terman, 2002). As in adult mice, microglia in aged mice show regional heterogeneity in the dynamics of their age-related changes in gene expression (Grabert et al., 2016; Masuda et al., 2019). Older animals exhibit increased mRNA levels of major histocompatibility complex (MHC) class II, cluster of differentiation 86 (CD86), class II, major histocompatibility complex trans-activator (CIITA), and interferon gamma (IFN-γ). At the same time, molecules that limit macrophage activation, such as interleukin-10 (IL-10) and cluster of differentiation 200 (CD200) are reduced (Sierra et al., 2007). Studies on mouse and human have highlighted that microglia in the aged brain ultimately show alterations in genes related to immune functions (e.g., interferon signaling and immunoreceptor expression), cell-adhesion and phagocytosis (Olah et al., 2018; Hammond et al., 2019). It was shown that the blockade of CD22, a sialic acid binding receptor which is upregulated in microglia during aging, not only restored phagocytosis, but it also improved the cognitive function in aged mice (Pluvinage et al., 2019). Since microglia acquire a more pro-inflammatory state with age, they are “primed” and respond much stronger to any kind of second stimulus. As a result, pro-inflammatory cytokines are released more rapidly and to a greater extent (Henry et al., 2009; Salvi et al., 2017). Microglial depletion and repopulation partially reverse the age-related transcriptomic profile. However, in response to a secondary LPS stimulation, microglia from aged mice still show an exaggerated response compared to adult mice, which hints on the role of the microglial microenvironment and the permanence of the pro-inflammatory phenotype in the aged brain (O’Neil et al., 2018). These data are in accordance with the idea that the aging brain displays a continuing, low-level, pro-inflammatory state, which is termed “brain inflammaging.” In fact, inflammaging results from chronic, low-threshold activation of the innate immune system, which can lead to a damaging, pathological situation (Franceschi et al., 2000). Innate immune cell functions decline with aging (Shaw et al., 2013). As a consequence of this immunosenescence one could imagine that cell debris of dying cells will not get removed in the aging brain by brain macrophages and slowly builds up to create a cell damaging environment. This enrichment of senescent cells is indirectly the incentive for the development of a typical proinflammatory phenotype by increasing levels of interleukins (IL) like IL-1, IL-6, and IL-18 or tumor necrosis factor-α (TNF-α), which are only present in low-level. The continuous presence of cytokines appears sufficient to inflict neuronal damage and ultimately forms the basis for age-related neurodegeneration (Lalancette-Hebert et al., 2007). In support of this assumption, the overall health status and even the life span could be dramatically improved when senescent cells were eliminated from aged mice (Baker et al., 2011; Zhu et al., 2015). In line with the later, a steady increase in lipid-laden cells (LLCs) is observed in the aging CNS. Perilipin (PLIN), a lipid droplet-associated protein, is a marker for autophagolysosome formation and indicates an increased lipophagic activity. Distinctive from lipofuscin, non autofluorescent Oil Red^+^ PLIN^+^ microglia show a lipid-laden multilocular pattern. The lipid-laden microglia expressed senescence markers and showed elevated TNF-α production and disturbed autophagy (Shimabukuro et al., 2016). In addition, isolated microglia, which showed lipid droplet accumulation, had higher oxidative stress and NAD^+^/NADH ratio, implying metabolic defects. These lipid-accumulating microglia also elicited diminished phagocytosis, which was dependent on *GRN*, a gene that is implicated in frontotemporal dementia (Marschallinger et al., 2020). As microglia are environmental sensors that actively survey the CNS, age-related functional changes in microglia can be expected to also impact other CNS cells. For the senescent human brain it is not clear whether microglia are autonomously deteriorating or whether the observed morphological changes are merely a response to their changing microenvironment. But the age-related morphological changes most certainly affect other cells, especially neurons, which are directly contacted at their soma and synapses by microglia in response to the neuronal activation status (Lampron et al., 2013). They maintain brain homeostasis by responding rapidly to clear invading pathogens and tissue debris (Di Benedetto et al., 2017). It seems that the expression of several neuronal ligands, which can be sensed by microglial CX3C chemokine receptor 1 (CX3CR1) and CD200R receptors, decreases during the aging process and contribute to the sparking of a pro-inflammatory phenotype (Wu et al., 2015; Kabba et al., 2018). Moreover, microglia activation can lead to the secretion of soluble components that counteract apoptosis of oligodendrocytes, which is induced by TNF-α (Nicholas et al., 2002). Despite the age-related decline in surveillance function and the prominent activation of microglia, they still have the potential to upregulate genes promoting neuroprotection, secrete anti-inflammatory cytokines and regulate adaptive processes such as tissue repair (Bellver-Landete et al., 2019). The parallel modulation of both pro- and anti-inflammatory factors suggests that microglial cells are still able to self-regulate and restore homeostasis.

### CNS- or Border-Associated Macrophages

At CNS interfaces with the periphery, i.e., meninges, perivascular space, and choroid plexus, another population of macrophages exists, collectively referred to as border- or CNS-associated macrophages (BAMs or CAMs). BAMs/CAMs are not only distinguishable from microglia by morphology and location, but they also do not express the typical microglia markers, e.g., P2RY12 and TMEM119, yet, they show high levels of the class B scavenger receptor CD36 and the mannose receptor CD206. Similar to microglia, studies have shown that BAMs/CAMs subsets share a yolk sac origin, are long lived and exhibit only minor replenishment by circulating peripheral progenitors, except for dural macrophages and stromal macrophages. This choroid plexus subset of macrophages is postnatally substituted by bone marrow-derived progenitor cells (Kierdorf et al., 2019). Despite the distinctive location within specialized anatomical structures, thorough investigations have only recently started to unravel the heterogenous subset identities and physiological functions of BAMs/CAMs (Van Hove et al., 2019). For example, in the brain meninges, the pia matter predominantly harbored Lyve1^+^MHCII^+^ BAMs/CAMs, while subsets residing in the dura matter lacked Lyve1 expression (Mrdjen et al., 2018). At the interface between the bloodstream and the parenchyma, perivascular and meningeal macrophages monitor and filter the cerebrospinal fluid (CSF), and scavenge antigens and possibly harmful substances (Serrats et al., 2010; Nayak et al., 2012). Indeed, recent studies have underscored the potential role of macrophages along the dural sinuses in antigen presentation and immune surveillance of the lymphatic drainage. Here, it is important to note that in old animals the lymphatic drainage of CNS antigens was decreased in relation to younger animals (Louveau et al., 2018; Rustenhoven et al., 2021), yet the role of BAMs/CAMs in this process is unclear. BAMs/CAMs display specific alterations in the aged brain, which are associated with a heightened proinflammatory cytokine profile and antigen presentation to T cells. The number of CD38^+^MHCII^+^CCR2^–^ and CD38^–^MHCII^+^CCR2^–^ subsets increased in aged mice, while CD38^+^MHCII^–^CCR2^–^ and CD38^–^MHCII^+^CCR2^+^ subsets decreased (Mrdjen et al., 2018). Studies have proposed that the interaction between perivascular macrophages and other cellular components of the blood brain barrier (BBB) could regulate the BBB integrity and vascular permeability. Perivascular macrophages secrete vascular endothelial growth factor (VEGF) to regulate glucose uptake by brain endothelial cells (BECs) (Jais et al., 2016). BECs, key players of the BBB, are sensitive to age. BECs, specifically the capillaries, undergo age-related transcriptional changes, which are dependent on the age identity of the circulatory proteins and have known associations with brain aging and AD (Chen et al., 2020).

### Neutrophils and Dendritic Cells

Under homeostatic conditions, additional innate immune cells present at the interface of the CNS include neutrophils, and two DC subsets: classical (cDCc) and plasmacytoid (pDCs). Despite being restricted by the BBB from the brain parenchyma and CSF, neutrophils and DCs were shown to be modulating the CNS in the course of different pathologies like infections, trauma, autoimmune disorders or neurodegeneration. Neutrophil infiltrates secrete free oxygen radicals (ROS) and proteolytic enzymes (i.e., MMP-9) that damage the BBB and potentially injure neurons (Manda-Handzlik and Demkow, 2019). DCs are the major antigen presenting cells in the CNS due to the expression of MHCII. Upon inflammation, resident DCs are augmented with monocyte-derived DC (moDC), which differentiate from infiltrating Ly6C^*hi*^ monocytes and can be detrimental for the CNS. Depletion or inactivation of moDCs ameliorates clinical disability in mouse models of Multiple sclerosis (Giles et al., 2018). In aged mice, the frequency of neutrophils had high variations, but pDCs showed a robust drop. Moreover, the number of cDC subsets CD24^+^ cDC2s declined, while the amount of CD135^+^ cDC2s rose (Mrdjen et al., 2018). Detailed transcriptomic and functional studies on neutrophils and cDCs at the interfaces of the aging CNS are still lacking; however, based on their role in phagocytosis and antigen presentation, it is tempting to speculate on their involvement in the lymphatic drainage from the CNS.

### Innate Lymphoid Cells

Innate lymphoid cells (ILCs) are a rapidly expanding group of cells that are critical to the innate immune system. ILCs are tissue resident cells that show high organ-specific plasticity. These cells also stem from lymphoid progenitor as B and T lymphocytes and respond as one of the first cells to stimuli in their close vicinity but lack the ability to recognize antigens (Spits et al., 2013). ILCs are currently subdivided into three subclasses, which inherently mimic subsets of T-helper (Th) cells in their cytokine production and functions. The response of type 1 ILCs (ILC1s) is similar to Th1 cells. Parallel to Th2 cells, ILC2s produce cytokines like IL-4, IL-5, and IL-13, which typically contribute to allergic inflammations. When activated, ILC3s mimic Th17 cells by secreting IL-17 and IL-22 (Vivier et al., 2018). NK cells can be seen as cytotoxic ILCs and are very similar to ILC1s (both are CD3^–^NK1.1^+^) when it comes to phenotype and function. The main difference between ILC1s and NK cells can be found in proteins that contribute to cell retention into the tissues (i.e., CD49a, CD103, and the lectin CD69) (Romero-Suárez et al., 2019). Functionally, the peripheral role of ILCs has been profoundly investigated in the last decade. In particular, their immune response against allergens, viruses, and inflammation of the lung and intestine was carefully characterized (Vivier et al., 2018). ILC1s are mainly found in the choroid plexus of the brain, in meninges and within the brain parenchyma where only few NK cells are localized. ILC2s are long-lived but less abundant and are present in the meninges and the choroid plexus but are absent in the brain parenchyma (Gadani et al., 2017). In the aged brain, ILCs elicited a shift toward an ILC2 transcriptional profile and showed an increased abundance of ILC-like cells, which express a T memory stemness (T_*scm*_) signature (Golomb et al., 2020). Interestingly, through the reduction of TNFα and the enhancement of hippocampal neurogenesis, the intracerebroventricular transfer of activated ILC2 or the direct administration of IL-5, a major ILC2 product, clearly improved the learning and memory abilities in old mice (Fung et al., 2020). The recent findings on ILCs highlight one avenue where the innate immune cells in the CNS counteract the age-associated functional decline.

Immune surveillance systems work together, to keep the brain in a state of homeostasis. The expansion of neurological injury, which leads to neurodegeneration, may be favored by the endogenously advancing regression in immune effector activity. Furthermore, the CNS-resident and -associated innate immune cells’ constant immune surveillance makes them an important communication-axis between the microbiota and the CNS.

## The Aging Gut Microbiome

Both the host and the microbiota undergo age-related changes, and host–microbiota interactions may represent a pivotal component in aging. In several studies the analysis of numerous stool samples indicated that the overall composition but also the diversity of the microbiota found in the gut changes with age (Claesson et al., 2011; Lakshminarayanan et al., 2014). Indeed, a healthy microbiota could support human longevity (Badal et al., 2020), and a young-microbiota transplant could prolong the lifespan in fish (Smith et al., 2017). Although the current dogma holds that the gut microbiota of aged individuals becomes less diverse and variable with advancing age (Claesson et al., 2011). It is important to point out that changes in the microbiota of the elderly may derive from dietary changes, prolonged intestinal transit times, lack of physical activity, residency in elderly homes, hospitalization, recurrent infections and frequent use of antibiotics and other medications. One study showed that these changes in diversity do not depend on the chronological age but are rather influenced by biological or functional age (Maffei et al., 2017). On the other hand, a study on a Japanese population, which included subjects ranging from new-born babies to centenarians, has shown an age-dependent increase in the microbiota diversity that only dropped in the centenarian group (Xu et al., 2019). This leaves the link between microbiota diversity and aging obscure. Vicissitudes in the microbiota during aging are certainly causally connected to inflammaging, as a lasting activation of the immune system contributes to immunosenescence. A prolonged state of inflammation could make the host more susceptible to potentially disadvantageous bacteria, which in turn contribute to the progression of various pathological conditions in older adults (Bischoff, 2016). It should be mentioned that in this context the human gut ecosystem is the best-studied microbiome. The reason is its ubiquitous role in converting environmental signals like nutrients from food into bioactive compounds that send signals to other organs including the brain. These bioactive compounds enable gut bacteria to interact and potentially modulate the function of the hosts‘ central nervous system. One can imagine that this kind of interface is not only formed by the microbiota of the gut. There are also reports on the aging microbiome in the lung, vagina, and urogenital tract and the possibility that bacteria from these niches have an indirect impact on brain function (Janiak et al., 2021; Wilmanski et al., 2021). To interpret these results, it seems that with increasing biological age, the diversity of gut microbiota shifts and at the same time specific microbial taxa emerge that are linked to unhealthy aging.

## How Gut Microbiota Interferes With Innate Immune Cells of the Aged Brain

The interaction between the gut microbiota and the innate and adaptive immune systems through direct engagements at mucosal surfaces or microbiota derived metabolites is unambiguous. The peripheral immune system is quite sensitive to slight alterations in the circulating metabolites and plasma cytokine composition, which can result due to microbiota dysbiosis (Zheng et al., 2020). Intriguingly, parabiosis or plasma transfer experiments that expose a young animal to old blood decreases hippocampal neurogenesis, promotes microgliosis and, ultimately, impairs learning and memory function (Villeda et al., 2011). On the other hand, exposing aged animals to young blood improves the cerebral vasculature, enhances neurogenesis in the subventricular zone and ameliorates the decline in olfaction (Katsimpardi et al., 2014). The brain has long been thought to be immune-privileged. However, the test of time has proved this terminology not absolute. Under homeostatic conditions, the degree of immune-privilege varies depending on age and neurological health. Additionally to the aforementioned age-associated alteration of the microbiota in aging, the neurovascular unit of the BBB undergoes a transition in caveolar transcytosis from ligand-specific receptor-mediated to a non-specific mode, which could potentially allow atypical primary or secondary microbiota-derived molecules uptake into the CNS (Yang et al., 2020). Indeed, beyond peripheral immunity, microbiota-derived signaling molecules have been implicated in CNS immunity, neuropsychiatric and neurodegenerative disorders (Mossad and Erny, 2020).

Compared to other understudied CNS innate immune cells, the microbiota-microglia axis has been well investigated during development and adulthood (Mezö et al., 2019). In a time-dependent and sexually dimorphic manner, germ-free (GF) mice, acute antibiotic intervention, or a lack of taxonomic sophistication hinder the development and immune response of microglia (Erny et al., 2015; Thion et al., 2018). Recolonization with complex microbiota or dietary supplementation with short-chain fatty acids (SCFAs) seems to reverse these defects, as activation of the SCFA receptor FFAR2 restores microglial maturation (Erny et al., 2015). These findings indicate that maintaining microglia homeostasis requires a low level of subclinical immune activation through the microbiota. In line with previous reports, a recent study on aged mice has shown that microglia and BAMs/CAMs in aged mice shift toward a pro-inflammatory and antigen presentation phenotype, however, when antibiotics were applied for only a short time period, gut-dysbiosis had little impact on microglia and BAMs/CAMs homeostasis (Golomb et al., 2020). Another interesting finding on the interaction of the microbiota with macrophages during aging comes from a study on peripheral macrophages. Fecal microbiota transplantation (FMT) from aged mice to young GF recipients elevates circulating inflammatory cytokines and impairs macrophage phagocytosis (Thevaranjan et al., 2017). Furthermore, for ILCs, antibiotics treatment specifically reduced T_*scm*_-like ILCs transcriptome signature only in the aged mice (Golomb et al., 2020). Nevertheless, the functional relevance of the T_*scm*_-like ILCs in the aging brain remains unclear. Furthermore, the gut microbiota regulates IFNγ expression in meningeal NK cells. Under neuroinflammatory conditions, the IFNγ^+^ NK cells maintain a subset of astrocytes (LAMP1^+^TRAIL^+^), which reduces inflammatory processes in the CNS by initiating T cell apoptosis (Sanmarco et al., 2021).

### Age-Associated Gut Borne Metabolites

Since the diet, drug intake, lifestyle, and gut-microbiome composition all change as people age, the intestinal metabolic environment, or the levels of microbial metabolites, will eventually change as well.

#### SCFAs

Short-chain fatty acids (SCFAs) belong to the group of saturated fatty acids and result from fermented dietary fibers in the gut. Butyrate, propionate and acetate constitute the majority of SCFAs and have significant impacts on several physiological features and help to maintain the integrity of the gut barrier, they contribute to metabolism in the periphery and support gut homeostasis (Donohoe et al., 2011; Den Besten et al., 2013; Thorburn et al., 2014). Surprisingly, microbial signatures expressing high concentrations of SCFA producers have been found in many studies investigating the microbiota of normal and aged animals and humans (Kong et al., 2016), yet the fecal concentration of SCFAs decreases with age (Woodmansey et al., 2004). Other factors that can influence SCFA fecal levels include the carbon present in diet or the absorption/excretion of these metabolites, which may be impaired in older subjects. Low levels of bacterially derived SCFAs in the aged microbiota are partially to blame for the enhanced immune status and worse outcomes in aged mice after a stroke. SCFAs were restored to the level of the young microbiome by SCFA-producing bacteria (probiotics) and a food supply (prebiotics) for these bacteria, and stroke outcomes improved dramatically. Of importance, these effects on outcome were not related to the chronological age of the mice but to the age of the microbiome (Lee et al., 2020). As SCFAs were found to mimic features of the microbiota’s influence on microglia under homeostasis, the role of SCFAs on microglia in CNS disorders has recently gained increasing attention. For instance, GF-housed APP/PS1 transgenic mice that receive SCFAs show increased cerebral Aβ loads and a specific microglial activation that is caused by enhanced ApoE-Trem2 signaling (Colombo et al., 2020).

#### Lipids

As previously stated, dysregulation of lipid metabolism and intracellular accumulation of certain lipid moieties are hallmarks of microglia in the aged brain (Marschallinger et al., 2020). The microbiota has an effect on the lipid composition of metabolically vital organs including adipose tissue, liver, and lipid-rich organs like the retina, according to evidence from GF animals (Backhed et al., 2004; Orešič et al., 2009; Velagapudi et al., 2010). The brain displays high lipid content and a variety of different lipid categories (Han, 2007). With increasing age, the lipid composition of the brain is changing (Yu et al., 2020). Changes in cholesterol metabolism, for example, have been linked to age-related neurodegenerative disorders and cognitive impairment (Zhang and Liu, 2015). GF animals populated with an aged microbiota show changes in lipid profiles in the cortex and liver, which is intriguing (Albouery et al., 2020). Whether the aged-microbiota mediated dysregulation of lipids in the CNS extrapolates to the lipid-laden microglial phenotype in the aged brain or further affects other innate immune cells remains unexplored.

#### Trimethylamines

Dietary cholines are metabolized in the gut to form trimethylamines. In a first step, choline is broken down into trimethylamine (TMA), which is transported to the liver where it is converted into trimethylamine-N-oxide (TMAO) (Bennett et al., 2013). Increased plasma levels of TMAO affect cholesterol metabolism and are linked to a higher risk of several age-related diseases including cardiovascular disease, colorectal cancer and atherosclerosis (Koeth et al., 2013; Bae et al., 2014). Since TMAO is found in the brain it is most likely able to cross the BBB (Del Rio et al., 2017). While controlling for age, sex and APOE ε4 genotype, higher CSF levels of TMAO were detected in AD and mild cognitive impairment patients compared to healthy controls suggesting the potential involvement of TMAO in AD (Vogt et al., 2018). Moreover, plasma TMAO levels, in an aging cohort, were inversely correlated with cognitive functions through promoting pro-inflammatory signaling in astrocytes and microglia (Brunt et al., 2021).

#### Amino Acid Metabolites

Human metagenomic studies suggest the gut microbes to be largely involved in amino acid metabolism. Aromatic amino acids, i.e., tryptophan, are majorly supplied in humans through diet or produced by the microbiota. By stimulating astrocytes, bacterial conversion of tryptophan to indoles, ligands for the aryl hydrocarbon receptor (AhR), can reduce brain inflammation and restrict disease severity in MS mouse models (Rothhammer et al., 2016). AhR signaling in microglia fine-tunes the microglial expression of TGFα and VEGF-B, which in turn should regulate astrocyte activation (Rothhammer et al., 2018).

## Conclusion and Future Perspectives

There is an evident gap in understanding the direct and indirect links between the microbiota and CNS innate immune cells other than microglia. This gap is even wider when it comes to investigating these interactions in the context of aging. Studies utilizing GF mice, acute antibiotic treatments, and fecal microbiota transplantations from the different ages, can be combined with different genetic models or innate immune cell pharmacological targeting approaches in order to gain some insights into the age-associated impact of the microbiota. Combinations with deep NGS approaches are essential to understand the specific phenotype alterations that occur in the CNS innate immune cell populations upon microbiota dysbiosis. Moreover, extensive unbiased analyses of the microbiota-derived metabolites and peptides at different sites enroute to the CNS are required to understand the dynamics of these molecules and their specific effects on CNS functions. More importantly, major studies on aging human populations expanding on the integrative human microbiome project (Proctor et al., 2019), which dwells into the associations between the microbiota’s identity and the changes in circulating molecules in blood and CSF, can provide the translatable link between the microbiota-derived signaling molecules and the innate immune cells of the CNS.

In conclusion, it is difficult to comprehend the biological and molecular basis of senescence, as well as the interplay between microglial senescence and the gut microbiota regulating various functions in the healthy and diseased brain. This, however, represents a therapeutic opportunity that could lead to the discovery of new pharmacological targets for maintaining or restoring physiological tasks in long-lived individuals.

## Author Contributions

Both authors listed have made a substantial, direct and intellectual contribution to the work, and approved it for publication.

## Conflict of Interest

The authors declare that the research was conducted in the absence of any commercial or financial relationships that could be construed as a potential conflict of interest.
